# Monitoring Cell Activation and Extracellular Vesicle Generation in Platelet Concentrates for Transfusion

**DOI:** 10.3390/ijms252111577

**Published:** 2024-10-28

**Authors:** Ana Kolenc, Maja Grundner, Irma Hostnik, Elvira Maličev

**Affiliations:** 1 Blood Transfusion Center of Slovenia, Šlajmarjeva 6, 1000 Ljubljana, Slovenia; ana_kolenc@ztm.si (A.K.);; 2 Biotechnical Faculty, University of Ljubljana, Jamnikarjeva ulica 101, 1000 Ljubljana, Slovenia

**Keywords:** platelet storage, platelet activation, extracellular vesicles, imaging flow cytometry, nanoparticle tracking analysis

## Abstract

Platelets play a crucial role in blood transfusions, and understanding the changes that occur during their storage is important for maintaining the quality of preparations. In this study, we examined key alternating factors, with a particular focus on platelet activation and the release of extracellular vesicles. Additionally, we compared two detection methods—imaging flow cytometry (IFC) and nanoparticle tracking analysis (NTA)—for their effectiveness in detecting particles. Platelet concentrates were prepared by pooling buffy coats from five blood group-compatible donors in an additive solution. Samples were analysed after one, three, and seven days of storage for residual white blood cells (WBCs), glucose levels, platelet activation, and extracellular vesicle concentrations. Over the storage period, the total platelet concentration decreased slightly, while the residual WBC count remained stable. Glucose levels declined, whereas platelet activation and extracellular vesicle concentration increased, with a positive correlation between the two. The particle size remained relatively unchanged throughout the storage period. Ultimately, despite controlled processing and storage conditions, platelet activation, and the release of extracellular vesicles still occurred, which may have implications for transfusion recipients. Although an optimised method is still needed, IFC has proved to be specific and potentially appropriate for detecting extracellular vesicles in transfusion preparations.

## 1. Introduction

When treating patients with thrombocytopenia, trauma, or undergoing major surgical procedures, platelet transfusions are essential. Platelets for transfusion can be collected through apheresis from a single donor or pooled whole blood from multiple (usually 4–6) donors to create platelet concentrates [1]. The maintenance of platelets’ functional integrity throughout storage, which is crucial for their therapeutic efficacy [2], and quality control of blood products remain key priorities of blood transfusion activities. Parameters include cell counts and final unit volume of transfusion preparations, as well as the obligatory absence of viral and bacterial contaminants, antibodies, and certain metabolic products in the donor’s whole blood. Recent developments in biological and biochemical sciences have introduced additional methods for monitoring blood units, helping to prevent various potential adverse effects following transfusion [1].

Despite careful storage, collected platelets have a short shelf-life [3] and must be stored under conditions that minimise biochemical, structural, and functional alterations [4,5]. Platelet concentrates are stored at 20–24 °C in gas-permeable plastic bags with constant gentle agitation [1,3]. Although this temperature helps maintain viability, it also promotes bacterial growth, limiting the standard storage duration to five days [3]. In blood transfusion centres, where bacterial contamination reduction methods, like psoralen, are used, this period may be extended to seven days.

Over time, platelets become activated and release extracellular vesicles [4,5]. This can also be influenced by stress from the blood collection or preparation of platelet concentrates [6]. These extracellular vesicles can impact various cellular processes and potentially negatively affect transfusion recipients [7]. On the other hand, the amount of extracellular vesicles in platelet concentrates could serve as an indicator of platelet activation, providing valuable information about the state of the preparation.

Prolonged storage of platelet concentrates can reduce transfusion effectiveness and increase adverse outcomes, such as immune-mediated reactions and inflammation, in addition to transfusion-associated sepsis [3]. Finding a balance between reducing waste and ensuring safety and quality is critical in determining the optimal storage duration, but extending the expiry date of platelets is the desired goal [8]. While some studies, like Aubron et al. (2018), suggest that storage within the approved period does not affect clinical and transfusion outcomes [3], others, like Caram-Deelder et al. (2016), show that fresher platelets are superior [8]. Neither study fully explored the altered state of the stored platelets.

After prolonged platelet storage duration, intra-bag platelet activation has already been demonstrated [2,9,10]. Activated platelets display changed shape and surface marker expression and decreased haemostasis effectiveness [2,5,9,10,11]. Markers that can determine platelet activation are different cytokines and chemokines (PF4/CXCL4, CXCL5, CXCL7, CXCL8, RANTES, etc.), adhesion molecules (P-selectin, fibrinogen, integrins), coagulation factors, and granule membrane-specific proteins (CD63) [12]. Platelets are easily stimulated cells, and when activated, they form and release large quantities of extracellular vesicles, which are cleared by monocytes/macrophages and other leucocytes. Residual white blood cells (WBCs) in platelet concentrates are another concern. These cells can provoke adverse reactions associated with cell immunomodulation and alloimmunisation to human leukocyte antigen (HLA) in graft versus host disease (GvHD) [13,14]. Reducing leukocyte levels has been shown to lessen transfusion-related complications [15].

Glucose levels in storage solutions also play a pivotal role in maintaining platelet metabolism. As platelets absorb glucose, reduced glycolysis by-products accumulate, leading to a pH drop, negatively impacting platelet viability and activation [1]. According to the European Directorate for the Quality of Medicines & HealthCare (EDQM) guidelines (2023), glucose levels measured at the end of the storage period must be above the limit of quantification or the pH has to be higher than 6.4 [1]. Additionally, extracellular vesicles, carrying pro-inflammatory and procoagulant factors [16], are released and can further stimulate platelet activation and could have an impact on transfusion recipients [6,17].

The main focus of our research was to assess the impact of storage duration on platelet activation, extracellular vesicle concentration, and their size. While increased platelet activation and decreased glucose levels during prolonged storage have already been demonstrated, we aim to emphasise our contribution by highlighting the elevated concentrations of extracellular vesicles over time, compromising the quality of platelet transfusions. Different techniques for the quantification of extracellular vesicles are being used, such as dynamic light scattering (DLS), resistive pulse sensing (RPS), indirect methods like total protein or nucleic acid quantification, imaging flow cytometry (IFC), nanoparticle tracking analysis (NTA), and flow cytometry. None of those are considered the gold standard; however, according to the MISEV guidelines, NTA and flow cytometry are the most suitable. Therefore, we employed these two methods for detecting extracellular vesicles in platelet concentrates [18,19,20].

## 2. Results

### 2.1. Platelet and Residual White Blood Cell Counts

**Figure 1:**
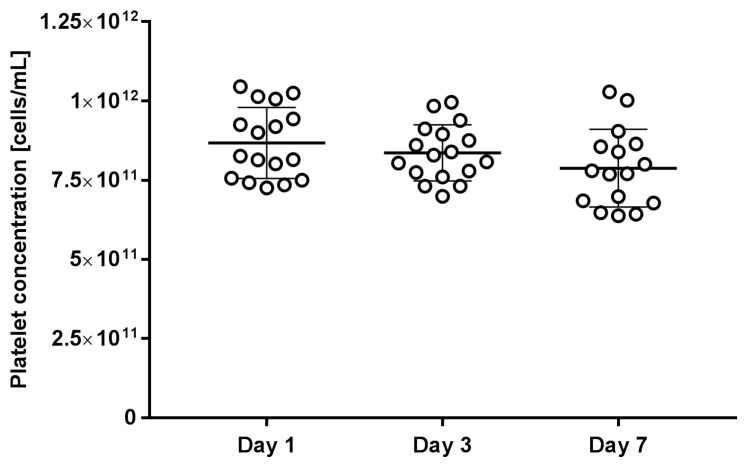
Platelet concentration in stored platelet concentrates on days 1, 3, and 7 after pooling. The figure shows individual measurements and average values with standard deviation of three independent experiments.

The platelet count per microliter in platelet concentrates was obtained by an automated blood cell counter. The average platelet concentration was 8.7 ± 1.1 × 10^11^ cells/mL after one day of storage, 8.4 ± 0.8 × 10^11^ cells/mL after three days of storage, and 7.9 ± 1.2 × 10^11^ cells/mL after seven days of storage. After analysing the results, we did not find a statistically significant difference between different storage days, although a small consumption of platelets during the storage period was detected (Figure 1). The platelet count after seven days of storage was still above the acceptable limit that requests a minimum of 2.0 × 10^11^ platelets per final unit prepared for transfusion [1].

**Figure 2:**
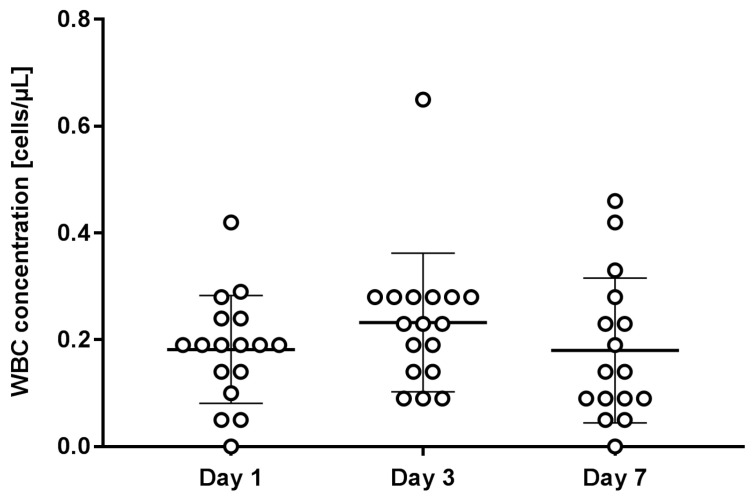
Concentration of residual white blood cells at different time points. Figure shows individual measurements and average values with standard deviation of three independent experiments.

In the same platelet concentrates, residual WBCs were detected. Figure 2 shows the number of residuals per microliter as a function of storage time. The presence of residual WBCs was measured in all 50 platelet concentrates. The measurements did not detect a statistically significant correlation between the content of residual WBCs and the storage time (r = −0.041). All the results met the standards with less than 1.0 × 10^6^ WBC per final unit [1].

### 2.2. Glucose Level

**Figure 3:**
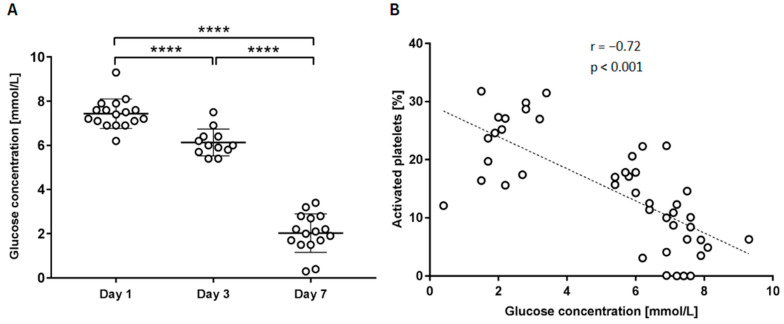
(**A**) Concentration of glucose at different time points. Figure shows individual measurements and average value with standard deviation of three independent experiments. Statistical difference is indicated with asterisks, **** *p* < 0.0001. (**B**) Correlation between glucose concentration and platelet activation. Figure represents the percentage of activated platelets compared to glucose concentration analysed using the Pearson Correlation and Student’s *t*-test (n = 44). An r value of 0.72 indicates a significant correlation with *p* < 0.001.

In total, 45 samples were analysed for glucose concentration: 17 samples after one day, 12 samples after three days, and 16 after seven days of storage. Figure 3 shows that the glucose concentration decreases with time as expected at an average of 7.44 ± 0.65 mmol/L on day 1, an average of 6.13 ± 0.58 mmol/L on day 3, to an average of 2.03 ± 0.84 mmol/L on day 7. A negative correlation was observed between glucose concentration and the percentage of activated platelets.

### 2.3. Platelet Activation

**Figure 4:**
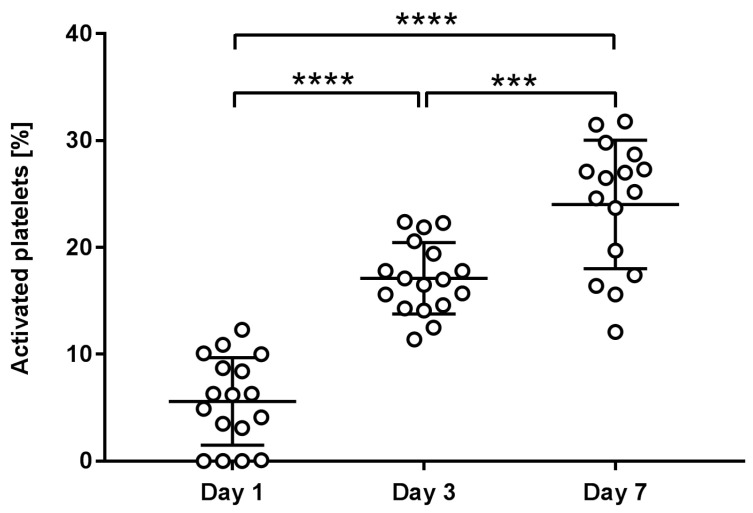
The percentage of activated platelets, identified by the presence of P-selectin on their surface, over a storage period of seven days. The figure shows individual measurements and average values with a standard deviation of three independent experiments. Statistical difference is indicated with asterisks, *** *p* < 0.001 and **** *p* < 0.0001.

**Figure 5:**
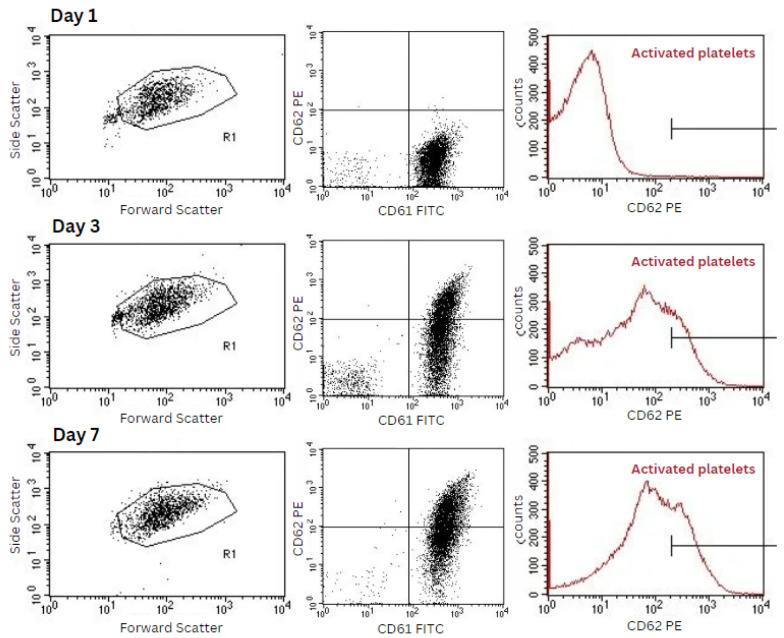
The gating strategy used on the FACSCalibur flow cytometer to determine the percentage of activated platelets (CD62P) among the total platelet population (CD61). Initially, platelets were gated based on their characteristic forward scatter (FSC) and side scatter (SSC) properties.

We assessed the percentage of activated platelets based on the presence of P-selectin on the platelet surface, which is expressed upon platelet activation. Our findings indicate that the proportion of activated platelets in the preparation increases over the storage period. After one day of storage, the percentage of activated platelets was 5.6 ± 3.9%, which increased to 17.1 ± 3.2% after three days and reached 24.0 ± 5.8% after seven days. A statistically significant difference was observed when comparing the percentages on the first, third, and seventh day (Figure 4 and Figure 5).

### 2.4. Concentration and Size of Extracellular Vesicles

**Figure 6:**
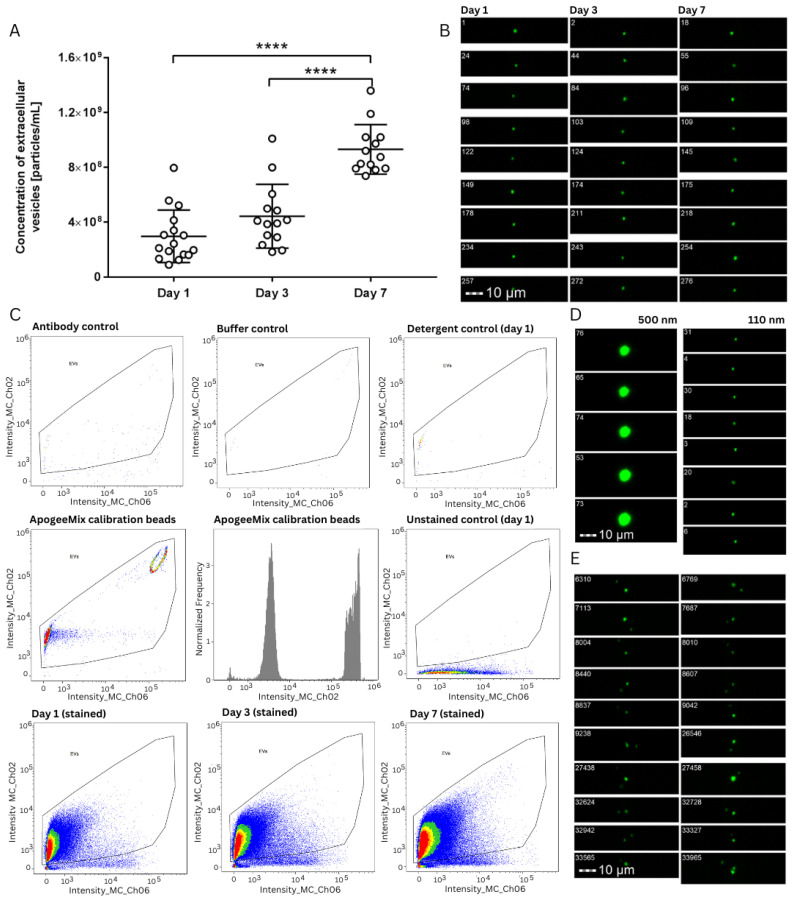
Results of analysis using ImageStream Imaging Flow Cytometer. (**A**) Concentration of extracellular vesicles over the storage time of platelet preparations. The figure shows individual measurements and average values with a standard deviation of three independent experiments. Statistical difference is indicated with asterisks, **** *p* < 0.0001. (**B**) Examples of CD9-positive events from days 1, 3, and 7 samples that correlate with events on respective scatterplots. (**C**) Gating strategy for the final positive results after excluding coincidence events and multiple events, along with events with high SSC. All the used controls and calibration beads are also included. (**D**) Examples of small and large fluorescent calibration bead populations used for the initial acquisition gate. (**E**) Examples of multiple events excluded from the analysis with appropriate masks and features of IDEAS software (Version 6.3).

**Figure 7:**
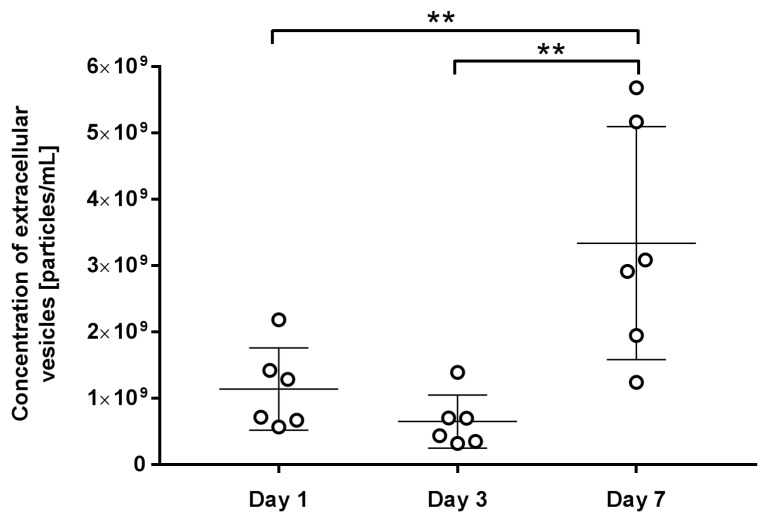
The concentration of extracellular vesicles, as measured by the NTA. The figure shows individual measurements and average values with a standard deviation of three independent experiments. Statistical difference is indicated with asterisks, ** *p* < 0.01.

The concentration and size of extracellular vesicles were analysed in duplicates using NTA after sample ultracentrifugation and ImageStream IFC. Extracellular vesicles were collected from 43 platelet concentrates, of which 17 were collected after one day, 13 after three days, and 13 after seven days of storage. Our data gained from the IFC and NTA measurements showed the same trend. In both experiments, a time-dependent rise in the concentration of extracellular vesicles during storage was revealed, with a statistically significant increase in vesicle concentration on the seventh day compared to the first and third days (Figure 6 and Figure 7 and Table 1).

Figure 6 shows the gallery of positive events and the gating strategy on the scatterplot of fluorescence against SSC for random samples of one, three, and seven days of storage. There were also controls included that showed the minimal presence of positive events in the gate. An unstained control was used for the appropriate gating and exclusion of negative events.

**Figure 8:**
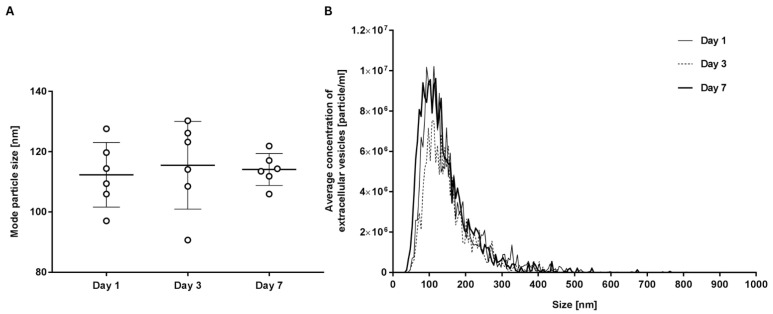
The size of extracellular vesicles according to NTA. (**A**) Figure shows individual measurements and average values with standard deviation of three independent experiments. (**B**) Example of the size distribution of extracellular vesicles of individual sample on days 1, 3, and 7.

Using NTA, the average size and size distribution of extracellular vesicles in each sample were measured. We demonstrated that different times of storage do not affect the average size of the vesicles (Table 1, Figure 8). Specifically, we determined the mode, which represents the particle size at the highest particle concentration. The results indicated that particle size did not change significantly throughout the storage period (Figure 8). Extracellular vesicles had an average size of 112.4 ± 9.8 nm on day 1, 115.5 ± 13.3 nm on day 3, and 113.4 ± 6.1 nm on day 7.

**Figure 9:**
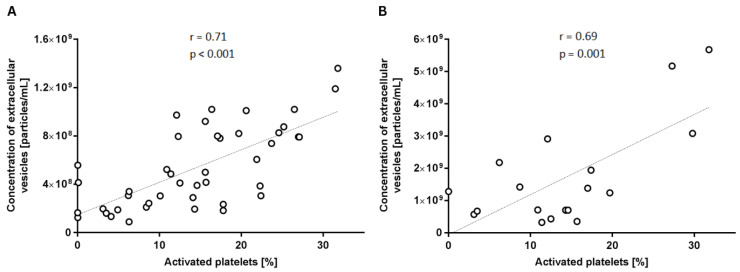
Correlation between the concentration of extracellular vesicles and platelet activation on ImageStream IFC (**A**) and NTA (**B**). The figure represents the percentage of activated platelets compared to the percentage of activated platelets analysed using the Pearson Correlation and Student’s *t*-test (n = 44 for A and n = 18 for B).

We investigated whether the proportion of activated platelets is associated with the concentration of extracellular vesicles in the samples. This correlation was assessed using two methods: NTA and IFC (Figure 9). Both analyses demonstrated a significant robust linear correlation between the concentration of extracellular vesicles and the proportion of activated platelets (*p* < 0.05), indicating that as platelets activate over time, they release more extracellular vesicles.

**Figure 10:**
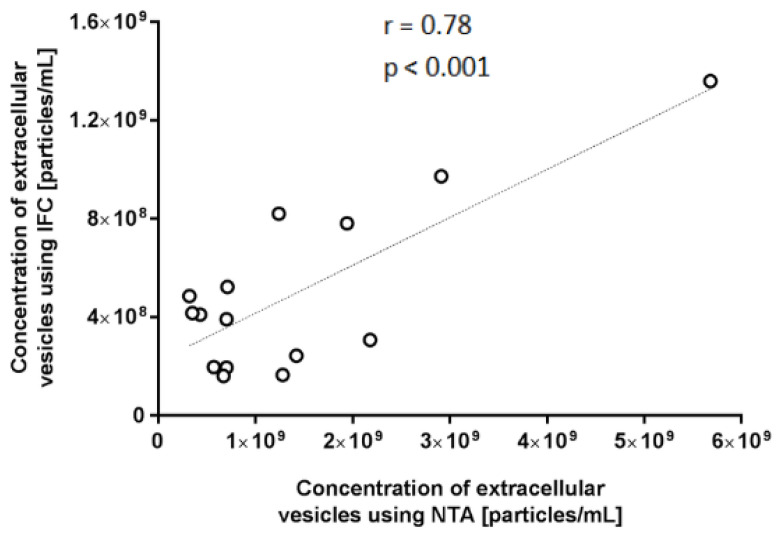
The concentration of extracellular vesicles measured by IFC and NTA. The correlation between the two methods was analysed using the Pearson Correlation and Student’s *t*-test (n = 18). An r value of 0.78 indicates a significant correlation with *p* < 0.001.

The relationship between the results gained with IFC and NTA is also shown in Figure 10. With a *p*-value of less than 0.001, the analysis produced a Pearson’s r of 0.78, which is evidence of a strong and statistically significant correlation.

## 3. Discussion

In our study, pooled platelet concentrates have been used, although preparations gained from aphaeresis could also be studied. The main reason for using pooled platelet concentrates was a relatively low number of aphaeresis donations collected during a routine practise at the Blood Transfusion Center of Slovenia. Pooled platelet concentrates are prepared from the platelets of five donors and harvested through whole blood centrifugation. The quality of these concentrates is crucial for safe and efficient transfusion; however, maintaining the platelet therapeutic efficacy during storage presents challenges. Factors such as platelet activation, glucose depletion, and the release of extracellular vesicles contribute to these difficulties. Platelets stored for transfusion are under strict regulations by the EDQM [1].

Research over the decades has revealed that prolonged platelet storage leads to a decline in therapeutic efficacy. Morphological, biochemical, and functional changes, including the release of cytosolic proteins, enhanced procoagulant activity, and altered expression of some glycoproteins, have been observed [21]. While some quality control parameters have been established for monitoring stored platelets, other indicators such as extracellular vesicle detection are still under investigation and are currently not a part of regular quality control [1]. Further, it is still not clear which technology and methods are most suitable for determining the size and amount of extracellular vesicles [18].

Our research aimed to assess the established and emerging quality parameters in stored platelet concentrates, including platelet activation and extracellular vesicle detection. Using two different methods, we compared the results for the extracellular vesicle concentration with other relevant parameters. The platelet samples were collected from five ABO-compatible whole blood donations, pooled, leukocyte-depleted, and pathogen-reduced using psoralen technology. Although storage lasted only seven days—the maximum allowed—this brief period still impacted the platelet concentrates. The platelet count decreased slightly, while the residual WBC count remained stable. The minimum standard for pooled platelet concentrates of 2 × 10^11^ platelets and fewer than one million leukocytes per unit [1] was met in our study.

Glucose and lactate concentrations can serve as indicators of platelet concentrate quality. Platelets are stored in a solution containing sodium acetate, sodium citrate, sodium phosphate, and magnesium chloride. The acetate and buffering salts help reduce glucose oxidation into lactic acid, thereby preventing significant pH drops [1]. The depletion of glucose and a rise in lactate above 28 mmol/L can result in low ATP levels and a rapid decrease in pH, which can impair platelet viability. More studies [22,23] showed a platelet glucose uptake linked to the phase of activation. Aerobic glycolysis, preferentially used for ATP generation while activating [22], accelerates flux through the pentose phosphate pathway and supports platelet activation [24]. Our results mirrored previous studies, showing a decrease in glucose concentration after seven days of storage (2.03 ± 0.84 mmol/L) (Figure 3A). Also, we observed a negative correlation between glucose concentration and the percentage of activated platelets (Figure 3B).

The increase in platelet activation during storage is also likely due to the combination of biochemical changes (accumulation of reactive oxygen species, decreased levels of glucose and ATP), physical stresses (mechanical stress, temperature fluctuations), and interaction with storage containers, the concentration of residual leukocytes, and the depletion of inhibitory factors in storage solution. Over storage, platelets’ integrity and functionality decline are directly connected to platelet activation, which occurs progressively during storage and leads to enhanced vesiculation [25]. In our study, the percentage of activated platelets increased from 5.6 ± 3.9% to 24.0 ± 5.8% over seven days of storage at 20–24 °C. A similar time-dependent increase in platelet activation has been reported in other studies, though the magnitude of activation varies depending on the method used [26].

The result of the platelet activation is also the formation of extracellular vesicles. Vesicles released from platelets can carry various biomolecules, such as proteins, lipids, and RNA, influencing a range of physiological and pathological processes, including haemostasis, coagulation, and immune responses [16,27,28,29]. The spontaneous release of extracellular vesicles can be explained simply as part of the platelet’s natural ageing and degradation process. The very collection and processing of blood before storage can accelerate vesiculation [30]. Vesiculation can be triggered by biochemical agonists such as collagen, thrombin, Ca^2+^, and lipopolysaccharide [16,27]; however, managing blood preparation conditions and shear stress have also been identified as factors that contribute to extracellular vesicle formation [6]. We observed an increase in the release of extracellular vesicles with storage time. When measured by IFC, the concentration increased from 2.97 to 9.32 × 10^8^ particles/mL. NTA showed a similar rise, from 1.14 to 3.34 × 10^9^ particles/mL. The results are not normalised, but we recognise that if significant differences in producing cell concentrations were present, they should be taken into account and the final concentration modified accordingly. We recognised that NTA is commonly used, and is perhaps the most frequently employed method, for studying extracellular vesicles from various sample types. However, we aimed to compare its effectiveness with the rising popularity of IFC, as light-scattering techniques like NTA are not specific to extracellular vesicles and may record co-isolated particles such as lipoproteins and protein aggregates [18,31]. The increase in vesicle concentration observed with NTA could be due to overestimations. For instance, Almeria et al. (2019) also noted that when studying extracellular vesicles from mesenchymal stem cells, NTA reported concentrations over three orders of magnitude higher than flow cytometry [31]. In terms of sample preparation, samples analysed with NTA underwent prior ultracentrifugation, while IFC was applied directly to PDP. Our comparison of these two methods in assessing extracellular vesicle concentrations showed a strong correlation, indicating that IFC may be a potential approach for quantifying vesicles in platelet concentrates. Given the specificity of flow cytometry for the selected markers on vesicles, we believe that this method is superior. Although we included a detergent control, confirming that most particles are indeed lipid-based vesicles, residual membrane fragments or alpha granules could still be included in the final particle concentration. Future work will need to involve broader phenotypic analysis with additional markers to confirm the presence of EVs, including potential CD9-negative populations. According to MISEV2023’s five-component framework, we will need to introduce at least one more specific marker associated with vesicles and one component of co-isolated structures (at least two positive and one negative marker). By demonstrating additional markers and incorporating other methods for vesicle detection and characterisation, such as electron microscopy, we will possibly be able to distinguish between whole vesicles and membrane fragments as well. Although we demonstrated a correlation between the methods, the consistently higher values observed in all samples using NTA suggest that IFC more effectively distinguishes extracellular vesicles from impurities.

While NTA is adaptable for particle quantification due to its cost-effectiveness and ease of use, its limited capabilities (primarily focusing on particle size and concentration) may necessitate the use of IFC for detailed phenotypic analyses. IFC also has broader cross-usability, as it can be employed in various contexts, from cell biology to immunology; therefore, many blood banks are already equipped with flow cytometers [32].

Our results, therefore, show that as platelets become more activated, they release more extracellular vesicles. This aligns with other research, which has also shown that platelet activation is inevitable during the preparation and storage of platelet concentrates, leading to activation, fragmentation, vesiculation, and degradation [30]. In the study of Black et al. (2017), the concentration of extracellular vesicles increased during storage in 86% of investigated platelet concentrates [30]. In our study, all samples from the seventh day of storage had higher extracellular vesicle concentrations compared to those from the first day. When comparing studies, we must consider differences in storage and analysis methods, particularly since Black et al. (2017) worked with apheresis-derived platelet concentrates and conventional flow cytometry [30].

Besides the extracellular vesicle concentration, we also analysed their size distribution throughout the storage period. The results showed that particle size remained relatively stable. The term ‘extracellular vesicles’ encompasses three main subclasses—exosomes, microvesicles, and apoptotic bodies—which differ in biogenesis and size. Exosomes are smaller (30–100 nm) than microvesicles (100–1000 nm), but the line between both groups is difficult to set. We encountered this issue when classifying vesicles by size, as the majority of vesicles in our samples ranged between 100 and 200 nm. It is important to note that we measured the size using NTA after concentrating the vesicles via ultracentrifugation, which could affect size distribution as some larger or smaller particles might be lost. Despite the increase in the concentration of extracellular vesicles, we did not observe significant changes in the mode of vesicle size or the overall size distribution across different storage days. This suggests that the vesiculation process most probably produces extracellular vesicles of a consistent size, regardless of the storage duration.

To conclude, our study revealed a statistically significant increase in the concentration of extracellular vesicles during the storage of platelet concentrates, as measured by both NTA and IFC. Whereby prior centrifugation is required and analysis is not accurate, the NTA method is more often used in extracellular vesicle research. But we think that the IFC choice is better, mainly due to the possibility of the specific labelling of vesicles. Additionally, we observed a positive correlation between the concentration of extracellular vesicles and the percentage of activated platelets. Given this information, future studies should explore the functional impact of extracellular vesicles on transfusion recipients and investigate the effects of different storage conditions and preparation methods across various centres.

We believe that establishing a reliable and reproducible method is one of the key foundations in a very new field, and it serves as the basis for any future clinical studies. Going forward, we aim to conduct a study on apheresis preparations, which would allow us to monitor additional variables such as donor sex, age, and demographic factors. We are aware that our study has some limitations, such as a relatively small sample number, which may impact the generalisability of our results. As techniques advance and larger studies are conducted, it will become clearer whether methods for determining extracellular vesicle concentration and phenotype could be used as part of the quality control of stored blood units.

## 4. Materials and Methods

### 4.1. Sample Collection

**Figure 11:**
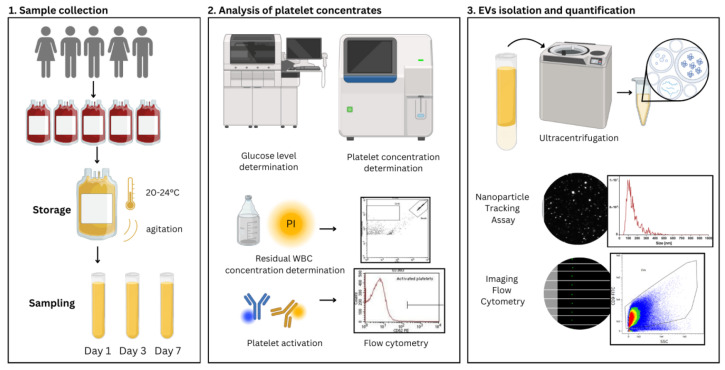
Study design.

Sample collection is a routine practise at the Blood Transfusion Center of Slovenia, and through this process, we have obtained the samples used in this study. Every blood donor satisfied the requirements set forth by regional and European standards (18–65 years old, adequate health and lifestyle, no exposure to infectious diseases, etc.). The European key criteria for blood and component donors, defined by Directive 2004/33/EC, Annex III, and Directive 2002/98/EC, which set the standards for the quality and safety of blood collection, testing, processing, storage, and distribution, were achieved [33,34]. For this study, five ABO blood group-compatible whole blood donations were used to create pooled leucocyte-depleted and pathogen-reduced platelet concentrates. Following the addition of the isotonic solution (CompoFlow Select^®^ blood bag system, Citrate Phosphate Dextrose Solution, Bad Homburg, Germany), they were stored in a final volume of 300–400 mL at a temperature of 20–24 °C under constant agitation. Following preparation, on days 1 (n = 17), 3 (n = 17), and 7 (n = 16), 50 samples of platelet concentrates were drawn under aseptic conditions, and activation and residual WBC were evaluated. All samples were analysed within an hour or stored at −80 °C (Figure 11). For other assays (analysis of extracellular vesicles using IFC and NTA), we had to prepare platelet-depleted plasma by two consecutive centrifugations of PC at room temperature at 2500× *g* for 15 min. Some analyses were not performed on all 50 samples because the samples were taken from preparations intended for potential transfusion, and we were unable to sample larger volumes.

We included all blood types in this study, with 30% representing blood group 0+, 30% A+, 12% B+, 8% AB+, 2% 0−, 8% A−, 6% B−, and 4% AB−.

The protocol of this study was approved by the Slovenian National Ethics Committee (0120-209/2020/3).

### 4.2. Determination of Platelet Concentration and Residual Leukocytes

Platelet concentrations were determined directly in the PCs using the CELL-DYN Ruby Haematology Analyzer (Abbott, Chigaco, IL, USA, LoD 0.99 × 10^9^ cells/L) using optical laser light scatter analysis. The concentration of residual leukocytes/white blood cells (WBCs) in the samples was determined by flow cytometry using a flow cytometer FACSCalibur (BD Biosciences, Erembodegem, Belgium) and a no-wash procedure. Residual leukocytes were stained with Leucocount human reagent (BD Bioscience, Franklin Lakes, NJ, USA) in Trucount tubes, containing a predefined number of counting beads (BD Biosciences, San Jose, CA, USA). Samples were prepared according to the manufacturer’s instructions. Briefly, 100 μL of platelet concentrates was dispensed into a Trucount tube with 400 μL of Leucocount reagent, gently vortexed, and incubated for 5 min in the dark at room temperature. After staining, samples were immediately analysed, and the concentration of residual WBC was determined using the following formula:

WBC/μL = total beads × WBC acquired/beads acquired × sample volume(1)

### 4.3. Determination of Platelet Activation in Platelet Concentrates

Platelet activation was measured with the detection of two markers: CD61 for all platelets and CD62p, also known as P-selectin, for activated platelets. Analysis was carried out immediately after sampling by flow cytometry using a flow cytometer FACSCalibur (BD Biosciences, Erembodegem, Belgium). In total, 10 μL of platelet-rich plasma was incubated in the dark at room temperature for 15 min with anti-CD61 FITC and anti-CD62p PE antibodies (BD Bioscience, Franklin Lakes, NJ, USA). After incubation, samples were fixed in 300 µL 1% paraformaldehyde and analysed, counting 100,000 CD61-positive cells (software CellQuest Pro 6.0, BD Bioscience, Franklin Lakes, NJ, USA). Activation of platelets was expressed as a percentage of CD62p+ events among CD61+ events. Each sample was labelled and measured in triplicate.

### 4.4. Glucose Level Determination

The glucose content in platelet concentrates after different storage times was determined on the clinical chemistry instrument Abbott Alinity ci^®^ Analyzer (Abbott Laboratories, Chicago, IL, USA) using the hexokinase method (LoD at 0.3 mmol/L). Glucose is phosphorylated by hexokinase and through several different reactions causes a reduction in nicotinamide adenine dinucleotide (NAD) to nicotinamide adenine dinucleotide (NADH). The produced NADH absorbs light at 340 nm. The increase in absorbance can be detected through spectrophotometry [35]. This diagnostic test is highly accurate and robust and is commonly used for measuring glucose concentration in biological fluids. We used platelet-depleted plasma samples that were stored at −80 °C; the preparation of these samples is covered in Section 4.1.

### 4.5. Enrichment of Small Extracellular Vesicles with Sucrose Cushion Ultracentrifugation

Platelet-depleted plasma was used for extracellular vesicles’ isolation after 1, 3, and 7 days after storage. First, the samples were additionally centrifuged at 10,000× *g* for 20 min at 4 °C for the removal of residual cells and larger debris. A total of 2 mL of 20% sucrose (Merk Millipore, Burlington, MA, USA) was pipetted in polypropylene tubes (Beckman Coulter, Brea, CA, USA) and topped with 0.9 mL of platelet-depleted plasma, which was diluted in 7.5 mL of particle-free Dulbecco’s phosphate-buffered saline (dPBS, Sigma-Aldrich, St. Louis, MO, USA) in advance. Samples were ultracentrifuged at 100,000× *g* for 135 min at 4 °C (MLA-55 rotor, Beckman Coulter, Brea, CA, USA). After the removal of the supernatant, the pellet containing enriched extracellular vesicles was resuspended in 60 µL of dPBS, and samples were stored at −80 °C. All enrichments were performed by the same person in the same laboratory.

### 4.6. Quantification of Extracellular Vesicles by Nanoparticle Tracking Assay (NTA)

Samples, enriched with extracellular vesicles, were diluted in dPBS to a concentration in the range of 107–109 EVs/mL and examined using a NanoSight NS300 (NanoSight Ltd., Amesbury, UK) equipped with a 488 nm blue laser. Five 60 s long videos were taken for each sample, with the best three chosen for analysis after visual inspection. Raw data of laser scattering and particle movement were analysed using NTA software (version 3.3; NanoSight Ltd., Amesbury, UK). Automatic settings were selected for the minimum expected particle site and blur, the minimum track length was set to 10, the detection threshold was set to 5, the sample viscosity sat set to the corresponding viscosity for water, and the temperature was set to 25 °C. The output data were presented as extracellular vesicles’ size (modal hydrodynamic diameter in nm) and the concentration of extracellular vesicles (number of extracellular vesicles enriched from 0.9 mL of plasma in particles/mL). Buffer control and standard were used to validate the accuracy of the measurements. Additionally, the results were normalised based on the standard.

All quantifications were performed by the same person in the same laboratory.

### 4.7. Imaging Flow Cytometry

ImageStreamX MkII Imaging Flow Cytometer (Cytek Amnis, Amsterdam, The Netherlands) equipped with 3 lasers (100 mW 488 nm, 150 mW 642 nm, and 70 mW 785 nm) and a 40× objective with NA of 0.75 and a DOF of 4 µm was used to analyse all the samples. All lasers were set to maximum power. Samples of platelet-depleted plasma were thawed, centrifuged at 2000× *g* for 20 min and diluted in dPBS to appropriate dilutions. All samples were stained with CD9-FITC (Miltenyi, Teterow, Germany; clone REA1071), previously centrifuged for 10 min at 12,500× *g*, and incubated for 2 h at room temperature. A titration experiment was performed to determine the appropriate final concentration of the antibodies used. Controls included for all analyses were (i) detergent lysis control (10% Triton X-100), (ii) buffer control without EVs (DPBS), (iii) antibody control (DPBS + antibody), and (iv) unstained samples. After staining, all samples were additionally diluted and prepared in duplicates. All data were acquired with 40× magnitude and a low flow rate for 10 min. FITC signals were collected in Channel 2 (480–560 nm). Channel 1 (430–480 nm filter) was used as the Brightfield channel and Channel 6 (745–800 nm filter) for SSC detection. dPBS was used as a standard sheath fluid. ApogeeMix Calibration beads (CAT #1527, Apogee Flow Systems, Castelldefels, Spain) were used to approximately set the initial acquisition gate based on the size and fluorescence of green fluorescent beads [36]. Data analysis was conducted using Amnis IDEAS software (Version 6.3), similar to the approach used in several other studies [37,38,39,40].

All quantifications were performed by the same person in the same laboratory.

### 4.8. Statistical Analysis

We utilised an analysis of variance (ANOVA) and Student’s *t*-test, with at least *p* < 0.05 considered significant. Additionally, we computed the correlation coefficient using Pearson’s coefficient to assess the strength and direction of the linear relationships between variables. For visualisation purposes, all graphs were generated using GraphPad Prism 7.03 software.

## 5. Conclusions

Despite carefully controlled processing and storage conditions, the changes in collected donor blood remain unavoidable. Our study aimed to examine certainly already well-known facts and the same factors which could be introduced into quality control of platelet concentrates. The results revealed a statistically significant increase in extracellular vesicles during the storage, as measured by both NTA and IFC, although we think the IFC method is more precise due to the possibility of the specific labelling of vesicles. Additionally, a positive correlation between the concentration of extracellular vesicles and the percentage of activated platelets is displayed. These findings have important implications for the clinical effectiveness of stored platelet concentrates, as increased activation and EV production may impact the haemostatic function of transfused platelets, potentially altering the therapeutic outcomes.

## Figures and Tables

**Table 1:** The average concentration of extracellular vesicles one, three, and seven days after storage according to imaging flow cytometry (IFC) and nanoparticle tracking analysis (NTA) and size of extracellular vesicles based on NTA.

Storage [Day]	Concentration on IFC [Particles/mL]	Concentration on NTA [Particles/mL]	Size [nm]
1	2.97 ± 1.86 × 10^8^	1.14 ± 5.66 × 10^9^	112.35 ± 9.79
3	4.44 ± 2.24 × 10^8^	6.48 ± 3.65 × 10^8^	115.5 ± 13.28
7	9.32 ± 1.81 × 10^8^	3.34 ± 1.6 × 10^9^	113.4 ± 6.14

## Data Availability

All data are given in the manuscript.
